# Feasibility and effectiveness of automatic deep learning network and radiomics models for differentiating tumor stroma ratio in pancreatic ductal adenocarcinoma

**DOI:** 10.1186/s13244-023-01553-z

**Published:** 2023-12-21

**Authors:** Hongfan Liao, Jiang Yuan, Chunhua Liu, Jiao Zhang, Yaying Yang, Hongwei Liang, Song Jiang, Shanxiong Chen, Yongmei Li, Yanbing Liu

**Affiliations:** 1 College of Medical Informatics, Chongqing Medical University, Chongqing, 400016 China; 2 Department of Radiology, the First Affiliated Hospital of Chongqing Medical University, Chongqing, 400016 China; 3 College of Computer and Information Science, Southwest University, Chongqing, 400715 China; 4 Department of Radiology, Daping Hospital, Army Medical University, Chongqing, China; 5 Department of Radiology, the Third Affiliated Hospital of Chongqing Medical University, Chongqing, China; 6 Department of Pathology, Molecular Medicine and Cancer Research Center, Chongqing Medical University, Chongqing, 400016 China; 7 Department of Radiology, Chongqing Ping An Medical Imaging Diagnosis Center, Chongqing, China

**Keywords:** Machine learning, Radiomics, Deep learning, Tumor stroma ratio, Pancreatic ductal adenocarcinoma

## Abstract

**Objective:**

This study aims to compare the feasibility and effectiveness of automatic deep learning network and radiomics models in differentiating low tumor stroma ratio (TSR) from high TSR in pancreatic ductal adenocarcinoma (PDAC).

**Methods:**

A retrospective analysis was conducted on a total of 207 PDAC patients from three centers (training cohort: *n* = 160; test cohort: *n* = 47). TSR was assessed on hematoxylin and eosin-stained specimens by experienced pathologists and divided as low TSR and high TSR. Deep learning and radiomics models were developed including ShuffulNetV2, Xception, MobileNetV3, ResNet18, support vector machine (SVM), k-nearest neighbor (KNN), random forest (RF), and logistic regression (LR). Additionally, the clinical models were constructed through univariate and multivariate logistic regression. Kaplan–Meier survival analysis and log-rank tests were conducted to compare the overall survival time between different TSR groups.

**Results:**

To differentiate low TSR from high TSR, the deep learning models based on ShuffulNetV2, Xception, MobileNetV3, and ResNet18 achieved AUCs of 0.846, 0.924, 0.930, and 0.941, respectively, outperforming the radiomics models based on SVM, KNN, RF, and LR with AUCs of 0.739, 0.717, 0.763, and 0.756, respectively. Resnet 18 achieved the best predictive performance. The clinical model based on T stage alone performed worse than deep learning models and radiomics models. The survival analysis based on 142 of the 207 patients demonstrated that patients with low TSR had longer overall survival.

**Conclusions:**

Deep learning models demonstrate feasibility and superiority over radiomics in differentiating TSR in PDAC. The tumor stroma ratio in the PDAC microenvironment plays a significant role in determining prognosis.

**Critical relevance statement:**

The objective was to compare the feasibility and effectiveness of automatic deep learning networks and radiomics models in identifying the tumor-stroma ratio in pancreatic ductal adenocarcinoma. Our findings demonstrate deep learning models exhibited superior performance compared to traditional radiomics models.

**Key points:**

• Deep learning demonstrates better performance than radiomics in differentiating tumor-stroma ratio in pancreatic ductal adenocarcinoma.

• The tumor-stroma ratio in the pancreatic ductal adenocarcinoma microenvironment plays a protective role in prognosis.

• Preoperative prediction of tumor-stroma ratio contributes to clinical decision-making and guiding precise medicine.

**Graphical Abstract:**

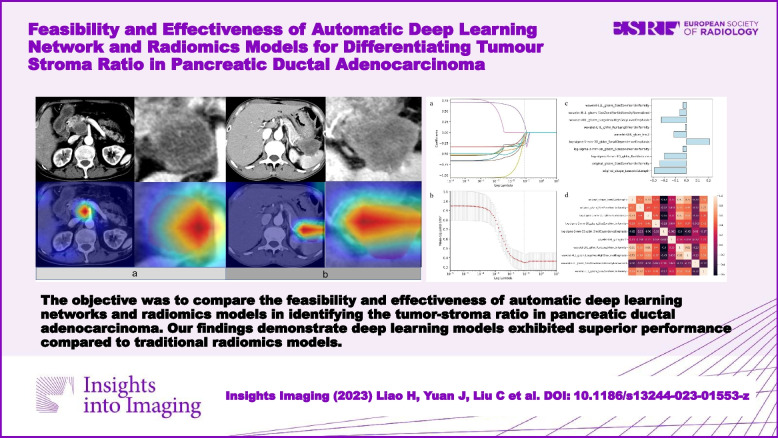

## Introduction

Pancreatic ductal adenocarcinoma (PDAC) has one of the most dismal prognoses among all human cancers, with a 5-year survival rate of approximately 9% [1, 2]. It is projected to become the second leading cause of cancer-related death in the coming decade [3]. Despite similar imaging manifestations and clinical stages, PDAC patients often exhibit significant variations in clinical outcomes [4, 5]. Traditional indicators alone are insufficient to predict prognosis accurately, necessitating the exploration of underlying biological characteristics to stratify patients based on their clinical outcomes.

The tumor microenvironment (TME) in PDAC is characterized by the presence of cancerous cells surrounded by desmoplastic and fibrotic stroma [6]. Previous studies have demonstrated that a high stromal content in PDAC patients plays a critical role in prognosis [7–9]. The tumor-stroma ratio (TSR), defined as the ratio of cancerous cells to the surrounding stroma [10, 11], has emerged as a significant indicator for evaluating disease progression in breast cancer, lung cancer, and gastric cancer [12–14]. Increasing evidence suggested low TSR is associated with longer postoperative survival, while high TSR is inclined to predict shorter survival and higher mortality [15, 16]. Additionally, an obvious improvement of prognosis after surgical resection was not observed in the high TSR group, and these patients must endure postoperative complications like pancreatitis and pancreatic fistula, which resulted in an adverse impact on quality of living [17, 18]. Emerging studies showed that the PDAC patients with more tumor-associated stroma result in the greater antitumor activity of hemotherapy agents or immune-mediated hypoxic necrosis of the tumor, who are more likely to benefit from interstitial targeted therapy [19]. Thus, the choice of treatment strategy may vary based on the distinct stromal composition of the tumor, and it is essential for clinicians to assess the stromal content prior to devising a more personalized and targeted therapeutic plan. However, obtaining TSR typically requires stained sections of surgical specimens, making it impractical for preoperative assessment. As a result, there exists a significant demand for the non-invasive and preoperative evaluation of TSR in cases of PDAC.

In recent years, machine learning techniques, including radiomics and deep learning, have shown tremendous potential in the field of medical imaging due to their reliability, high accuracy, and effectiveness in developing predictive models. Radiomics refers to extracting handcrafted features in a high-dimensional feature space from the region of interest (ROI) of radiographic images (CT, MRI, PET, etc.), and analyzing such image features (also known as biomarker) for accurate and quantitative evaluation of the lesions, and eventually used to assist in the diagnosis, classification of the disease. Deep learning as a new research direction in the field of machine learning, automatically learning complex features by combining lower-level features to form more abstract higher-level features. The advantage of deep learning is to replace manually designed hard-coded feature extraction used in radiomics [20–22]. With advancements in algorithms and artificial intelligence, several studies have explored the application of machine learning technology in PDAC [23–25]. Past studies have explored the correlation between radiomics features and TSR, constructing predictive models for TSR in PDAC [15, 16, 26]. Nevertheless, radiomics models come with their own set of limitations. In contrast, deep learning models have demonstrated superior ability in capturing the biological information revealed by CT images [27]. Nevertheless, few studies have constructed deep learning models for preoperative differentiation of TSR in PDAC patients [15, 16]. Therefore, the objective of our study was to compare the feasibility and effectiveness of automatic deep learning networks and radiomics models in identifying TSR in PDAC.

## Materials and methods

### Study population

**Fig. 1:**
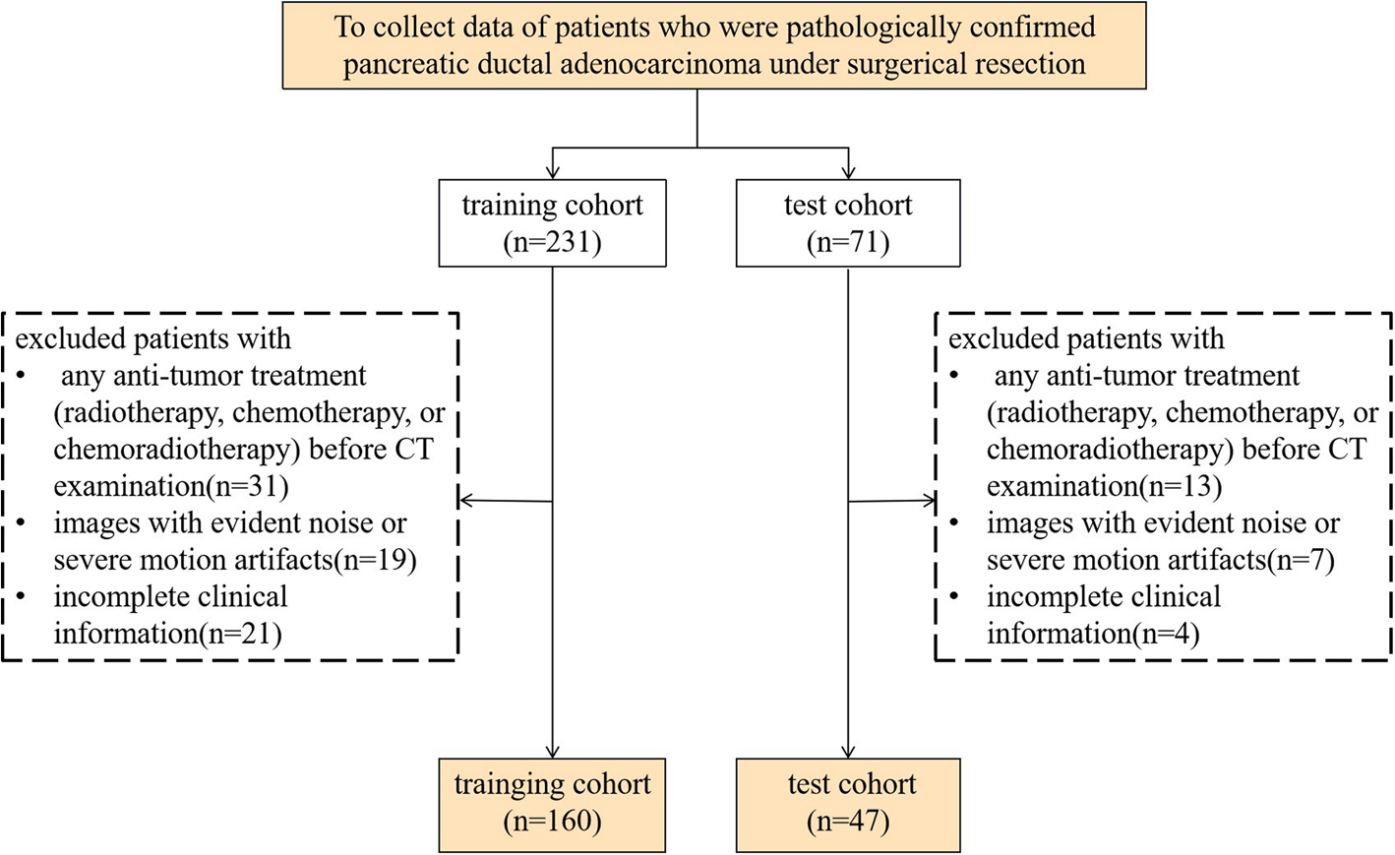
Flow chart illustrating the patient selection process

This retrospective study received approval from the local institutional review board (approval number: No.2022–63), and the need for informed consent was waived in accordance with the 1964 Helsinki declaration. The study was conducted using three tertiary referral hospitals in Chongqing Province. A total of 207 PDAC patients with confirmed pathology were recruited consecutively finally in the study. The training cohort (160 patients) was enrolled from the First Affiliated Hospital of Chongqing Medical University between 2013 Jan and 2021 Sep, and the independent test cohort (47 patients) was enrolled from Daping Hospital of Army Medical University between 2020 Sep and 2022 Jan and the Third Affiliated Hospital of Chongqing Medical University between 2021 March and 2022 June. The inclusion criteria were as follows: (1) patients who underwent surgical resection of the tumor, (2) availability of CT scans taken within 1 month before the surgery, and (3) visible pancreatic lesions on the CT images. The exclusion criteria were as follows: (1) patients who received any antitumor treatment (radiotherapy, chemotherapy, or chemoradiotherapy) prior to the CT examination, (2) images with noticeable noise or severe motion artifacts, and (3) incomplete clinical information. Due to the patients initially collected all underwent surgical resection, so PDAC patients with liver metastases and/or peritoneal carcinomatosis before surgery wouldn’t be enrolled for selection. The specific selection flowchart was displayed in Fig. 1. Baseline clinical data were collected from the electronic medical records system. Patients’ follow-up information was obtained through outpatient visits and telephone follow-ups. The overall survival time (OS) was defined as the interval between the date of operation and the date of death or the last known alive status.

### Imaging acquisition

A 128-slice multidetector-row CT scanner (SOMATOM Definition Flash, Siemens Healthineers) was used for the training cohort, and a 256-slice multidetector-row CT scanner (GE Revolution 256, GE Healthcare) and a 64-slice multidetector-row CT scanner (GE lightspeed vct) were used for the test cohort, respectively. Scans were performed in a craniocaudal direction, starting from the hepatic dome to the bilateral anterior superior iliac spine. The imaging protocol included an unenhanced phase, followed by the injection of a non-ionic contrast agent (Ultravist 350/370, Bayer Healthcare) at a specific dose (1.2 mL/kg) and flow rate (3.5–5.0 mL/s). A saline flush of 30–40 mL at the same injection rate was administered. The arterial phase scanning was initiated 10–15 s after reaching a trigger threshold (100 HU) in the abdominal aorta, and the portal venous phase scanning was conducted 30–35 s after the end of the arterial phase.

The acquisition parameters included tube voltages of 120 kVp, collimation of 128 × 0.6 mm (for Siemens scanner) and 64 × 0.625 mm (for GE scanners), gantry rotation time of 0.5 s, and spiral pitch of 1.0 (for Siemens scanner) or 0.7 (for GE scanners). All images were reconstructed with a thickness of 5 mm, an increment of 5 mm.

### Pathological image analysis

This was a retrospective process where the pathologists had access to specimens from a tissue bank in every hospital. The pathologists cut the entire specimens into 5-mm thick sections, generating 10–35 formalin-fixed paraffin-embedded (FFPE) blocks per specimen. Each FFPE block was sliced into 4 µm thick sections and stained with hematoxylin and eosin. A single field of moderate magnification (100 ×) was selected for analysis, ensuring that all four corners of the field of vision were within the tumor. The tumor-stroma ratio (TSR) was evaluated by quantifying the proportion of tumor and stroma components under microscopic examination. A TSR value of 5/5 was considered the optimal cutoff value based on previous studies [15, 16]. High stroma content was defined as TSR ≤ 1, while low stroma content was defined as TSR > 1. Based on the present observations, the TSR values were categorized into a low TSR group and a high TSR group. TSR evaluation was performed by two experienced pathologists, and a consensus was reached through joint evaluation in cases of disagreement in every hospital. In actuality, inconsistent observation between two pathologists was rare.

### Radiological imaging analysis

Image characteristics were assessed by two radiologists with 8 and 10 years of experience in abdominal imaging diagnosis, respectively, at a PACS workstation. Any discrepancies were resolved by consultation with the third radiologist (with 28 years of experience in abdominal imaging diagnosis). The baseline characteristics of all tumors were evaluated, including (1) clinical characteristics: age, sex, abdominal pain, pancreatitis history, and jaundice; (2) pathological characteristics: T stage, histological grade, lymph node metastasis, and duodenal invasion; (3) image characteristics: CT-reported tumor size, tumor location, parenchymal atrophy, pancreatic duct dilatation, and common bile duct dilatation; and (4) biochemical characteristics: carbohydrate antigen 19–9 (CA19-9) level, carcinoembryonic antigen (CEA) level, and total bilirubin (TBIL) level. Univariate and multivariate logistic regression analyses were performed on the above-mentioned variables. Ultimately, statistically significant features were selected for clinical model development.

### Radiomics workflow

**Fig. 2:**
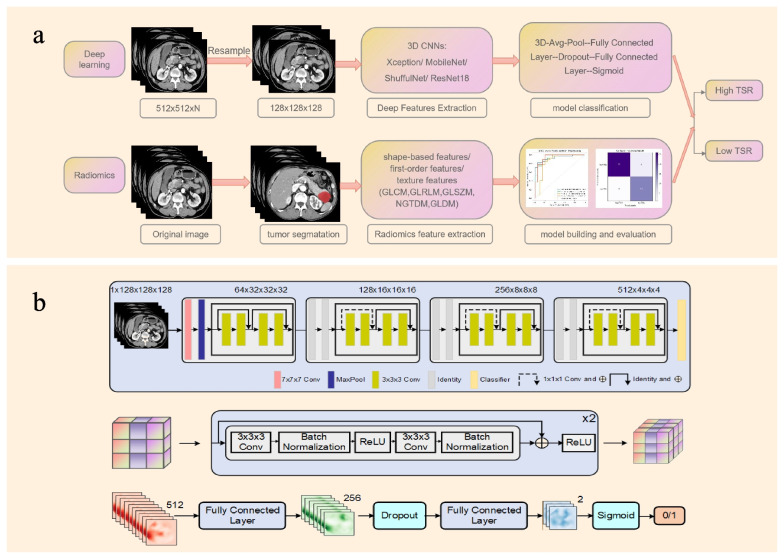
Workflow of this study and network structure of ResNet18. **a** The flowchart of this study. **b** Network structure of ResNet18 and the representative feature of shortcut connection

The standardized radiomics analysis workflow was employed following the Image Biomarker Standardization Initiative (IBSI) reporting guidelines [28]. (1) Tumor segmentation: Radiologist 1 performed three-dimensional volume of interest (3D-VOI) segmentation along the tumor margin excluding cysts, necrosis, blood vessels, and lymph nodes in side tumor on axial portal venous phase CT images using ITK-SNAP software (version 3.8.0, http://www.itksnap.org/). We did not choose the arterial phase because the tumor boundary was more distinct and evident in the portal venous phase, which contribute to tumor segmentation. To assess interobserver reliability, radiologist 2 conducted independent VOI delineations on the images of 30 randomly selected patients from both cohorts. One month later, radiologist 1 repeated the segmentation for 30 randomly selected patients who were different from 30 patients selected by radiologist 2 from both cohorts to evaluate intraobserver reliability. The inter- and intraobserver reliability was evaluated using the intraclass correlation coefficient (ICC), with ICC values > 0.75 indicating good consistency. (2) Feature extraction: Radiomics features, including shape features, first-order histogram features, and five texture features (gray level cooccurrence matrix (GLCM), gray level run length matrix (GLRLM), gray level size zone matrix (GLSZM), neighboring gray tone difference matrix (NGTDM), and gray level dependence matrix (GLDM)), were extracted using PyRadiomics 3.0 [29]. (3) Feature reduction and selection: analysis of variance, least absolute shrinkage, selection operator (Lasso) regression, and principal component analysis (PCA) were successively applied to screen and reduce the dimensionality of the features. The final selected features were normalized using a sigmoid function to ensure values between 0 and 1. (4) Radiomics model construction: In the selection of traditional radiomics models, we choose the representative of different machine learning algorithms, such as linear classifiers — logistic regression (LR), support vector machine (SVM); tree model-based algorithms — random forest (RF); classical clustering algorithms — K-nearest neighbor (KNN). Through these different machine learning algorithms, we evaluated which classification ideas were more suitable for this task. All models were constructed using five-fold cross-validation to avoid overfitting and ensure repeatability and reproductivity. A complete schematic is presented in Fig. 2a.

### Deep learning workflow

All original abdominal CT images, without any manual segmentation, were used in 3D format as input for the network. To enhance the robustness of the model and avoid overfitting, data augmentation strategies, including random clipping, random horizontal flip, and random vertical flip, were applied before feeding the images into the network. The loss function was calculated as the deviation between the output of the neural network and the label, and the weights of each layer were updated using the back-propagation algorithm. The best weights were determined based on the minimal loss value and fixed for subsequent use on the test cohort. Due to the small amount of experimental data, in the selection of the deep learning model, we chose representative lightweight deep learning model or networks with fewer parameters for experiments in deep learning: ShuffulNetv2, Xecption, MobileNetV3, and ResNet18 [30–32]. Specifically, the convolutional neural network has a good ability to extract local features, and this task requires the network to pay attention to local details of images, which is in line with the advantages of the convolutional neural network. Therefore, we chose a convolutional neural network to conduct the experiment. Next, our task is a coarse-grained prediction task with a small amount of data, and the use of a network with many parameters will result in serious overfitting and make it difficult for the network to learn effective information. Lightweight CNN models usually perform well on small data sets and are not easy to overfit because they are easier to generalize to previously unseen data. More complex models on small data sets may be more susceptible to noise or chance in the data. Therefore, we choose such four kinds of convolutional neural networks with fewer parameters and strong universality to conduct experiments, and further determine the networks more suitable for this task through experiments. These four pretrained 3D convolutional neural networks (CNNs) were used to construct end-to-end models. The AdamW optimizer with momentum parameters ${\mathrm\beta}_1=0.9\;\mathrm{and}\;{\mathrm\beta}_2=0.999$ was utilized, and the initial learning rate was set to 0.00001. CosineAnnealing was employed for learning rate decay. A total of 30 epochs were trained, with a penalty coefficient of 0.01, warm-up set to 1, batch size of 2, and dropout of 0.75. The experiments were conducted using python (https://www.python.org) and the pyTorch (https://www.pytorch.org) framework on an NVIDIA GeForce GTX 2080 SUPER GPU.

To enhance the transparency and interpretability of the model’s decision-making process, we applied gradient-weighted class activation mapping (Grad-CAM) to provide a visual explanation. Grad-CAM utilizes the gradient information from the last convolutional layer of the CNNs to obtain a class activation map. This map offered insights into the image regions that contributed most significantly to the model's classification and helped in validating its performance and identifying potential areas for improvement.

### Model evaluation and statistical analysis

The prediction performance of the radiomics and deep learning models was evaluated using various metrics, including the area under the curve (AUC), accuracy (ACC), precision, recall, and F1 score. The models’ performance was visualized using receiver operating characteristic (ROC) curves. Decision curve analysis (DCA) was used to quantify the net benefits with different threshold probabilities. Calibration curve analysis was employed to fit the actual and predicted incidence rates. The DeLong test was performed to compare the diagnostic efficiency among different models.

Quantitative variables between groups were compared using Student’s *t*-test if the distribution was normal, or the Mann‒Whitney *U* test if the distribution was non-normal. Qualitative variables between groups were compared using the chi-square test or Fisher's exact test. A *p*-value of less than 0.05 was considered statistically significant. SPSS software (version 23.0) was used for statistical analyses.

## Results

### Patient characteristics

In the training cohort, there were 72 (45%) patients in the TSR-low group and 88 (55%) patients in the TSR-high group. The independent test cohort consisted of 20 (43%) patients in the TSR-low group and 27 (57%) patients in the TSR-high group (Table 1). Significant statistical differences between the TSR-low and TSR-high groups were observed in the T stage (*p* = 0.048) in the training cohort and histological grade (*p* = 0.013) in the test cohort. No significant differences in any of the baseline characteristics were observed between training and test groups. After univariate and multvariate logistic regression, only the T stage (OR: 0.410, 95% CI: 0.205–0.821, *p* = 0.012) was retained for clinical model development (Table 2). The clinical model achieved an AUC of 0.566 (0.477, 0.654) in the training cohort and an AUC of 0.610 (0.448, 0.772) in the test cohort. Of the total 142 patients from the training cohort available for survival analysis (TSR-low: 69 patients, TSR-high: 73 patients), the Kaplan‒Meier curves demonstrated a significant difference (*p* < 0.05) between the TSR-high and TSR-low groups. The log-rank test indicated a significantly longer survival duration in the TSR-low group (mean: 25.81 months, 95% confidence interval [CI]: 21.39–30.23) compared to the TSR-high group (mean: 17.95 months, 95% CI: 14.28–21.62).

**Table 1:** Baseline characteristics in the training and test cohorts

Characteristics	Training cohort (*n* = 160)			External test cohort (*n* = 47)			*P* (Inter)
	TSR-low (*n* = 72)	TSR-high (*n* = 88)	*P* (Intra)	TSR-low (*n* = 20)	TSR-high (*n* = 27)	*P* (Intra)	
Clinical characteristics							
Age (years), mean ± SD	60.40 ± 9.45	60.55 ± 9.66	0.925	59.48 ± 9.04	59.85 ± 9.25	0.892	0.784
Gender			0.720			0.726	0.895
Female	24 (33.3)	27 (30.7)		5 (25.0)	8 (29.6)		
Male	48 (66.7)	61 (69.3)		15 (75.0)	19 (70.4)		
Abdominal pain			0.106			0.770	0.592
Yes	37 (51.4)	34 (38.6)		9 (45.0)	11 (40.7)		
No	35 (48.6)	54 (61.4)		11 (55.0)	16 (59.3)		
Pancreatitis history			0.302			0.640	0.611
No	55 (76.4)	73 (83.0)		16 (80.0)	23 (85.2)		
Yes	17 (23.6)	15 (17.0)		4 (20.0)	4 (14.8)		
Jaundice			0.806			0.380	0.457
No	56 (77.8)	67 (76.1)		15 ((75.0)	23 (85.2)		
Yes	16 (22.2)	21 (23.9)		5 (25.0)	4 (14.8)		
Pathological characteristics							
T stage			0.048*			0.095	0.301
T1–2	61 (84.7)	63 (71.6)		17 (85.0)	17 (63.0)		
T3–4	11 (15.3)	25 (28.4)		3 (15.0)	10 (37.0)		
Histological grade			0.829			0.013*	0.052
Low-grade	47 (65.3)	56 (63.6)		14 (70.0)	9 (33.3)		
High-grade	25 (34.7)	32 (36.4)		6 (30.0)	18 (66.7)		
Lymph node metastasis			0.423			0.638	0.413
Negative	43 (59.7)	47 (53.4)		12 (60.0)	18 (66.7)		
Positive	29 (40.3)	41 (46.6)		8 (40.0)	9 (33.3)		
Duodenum invasion			0.112			0.333	0.599
Negative	41 (56.9)	39 (44.3)		9 (45.0)	16 (59.3)		
Positive	31 (43.1)	49 (55.7)		11 (55.0)	11 (40.7)		
Imaging characteristics							
CT-reported tumor size	26.65 ± 11.31	28.57 ± 10.43	0.270	24.78 ± 17.31	29.95 ± 8.78	0.228	0.772
Location			0.173			0.905	0.850
Head and neck	50 (69.4)	73 (83.0)		13 (65.0)	18 (66.7)		
Body and tail	22 (30.6)	15 (17.0)		7 (35.0)	9 (33.3)		
Parenchymal atrophy			0.946			0.642	0.368
No	27 (38.0)	33 (37.5)		9 (45.0)	14 (51.9)		
Yes	44 (62.0)	55 (62.5)		11 (55.0)	13 (48.1)		
PD dilatation			0.493			0.635	0.859
No	18 (25.0)	18 (20.5)		4 (20.0)	7 (25.9)		
Yes	54 (75.0)	70 (79.5)		16 (80.0)	20 (74.1)		
CBD dilatation			0.607			0.381	0.914
No	24 (33.3)	26 (29.5)		5 (25.0)	10 (37.0)		
Yes	48 (66.7)	62 (70.5)		15 (75.0)	17 (63.0)		
Biochemical characteristics							
CA-199 level			0.167			0.260	0.774
Normal	27 (18.1)	24 (35.2)		2 (10.0)	11 (40.7)		
Abnormal	45 (81.9)	64 (64.8)		18 (90.0)	16 (59.3)		
CEA level			0.626			0.404	0.378
Normal	61 (84.7)	72 (81.8)		17 (85.0)	25 (92.6)		
Abnormal	11 (15.3)	26 (18.2)		3 (15.0)	2 (7.4)		
TBIL level			0.274			0.689	0.699
Normal	34 (47.2)	34 (38.6)		7 (35.0)	11 (40.7)		
Abnormal	38 (52.8)	54 (61.4)		13 (65.0)	16 (59.3)		

p(Intra) represents the result of univariable analyses between TSR-low and TSR-high groups, p(Inter) represents the significant difference between training and test groups

*PD* pancreatic duct, *CBD* common bile duct, *CA-199* carbohydrate antigen 199, *CEA* carcino-embryonic antigen, *TBIL* total bilirubin

^*^Represents *p* < 0.05

**Table 2:** Univariate and multivariable logistic regression analyses for selecting clinical features of model development

Characteristics	Univariate analysis		Multivariate analysis	
	OR (95% CI)	*p*-value	OR (95% CI)	*p*-value
Age	1.004 (0.972, 1.038)	0.793		
Gender	1.112 (0.575, 2.15)	0.753		
Abdominal pain	1.84 (0.944, 3.586)	0.074		
Pancreatitis history	0.612 (0.285, 1.314)	0.208		
Jaundice	0.822 (0.395, 1.708)	0.599		
T stage	0.416 (0.192, 0.902)	0.026*	0.410 (0.205, 0.821)	0.012*
Histological grade	1.729 (0.895, 3.342)	0.103		
Lymph node metastasis	1.102 (0.576, 2.111)	0.769		
Duodenum Invasion	1.32 (0.658, 2.648)	0.435		
CT-reported tumor size	0.978 (0.951, 1.006)	0.121		
Location	0.687 (0.331, 1.429)	0.316		
Parenchymal atrophy	0.835 (0.424, 1.646)	0.602		
PD dilatation	1.044 (0.452, 2.41)	0.92		
CBD dilatation	0.558 (0.227, 1.371)	0.203		
CA-199 level	1.281 (0.65, 2.526)	0.474		
CEA level	1.103 (0.467, 2.606)	0.824		
TBIL level	0.558 (0.227, 1.371)	0.149		

*OR* odds ratio, *CI* confidence interval, *PD* pancreatic duct, *CBD* common bile duct, *CA-199* carbohydrate antigen 199, *CEA* carcino-embryonic antigen, *TBIL* total bilirubin

^*^Represents *p* < 0.05

### Model performance based on radiomics and deep learning

**Fig. 3:**
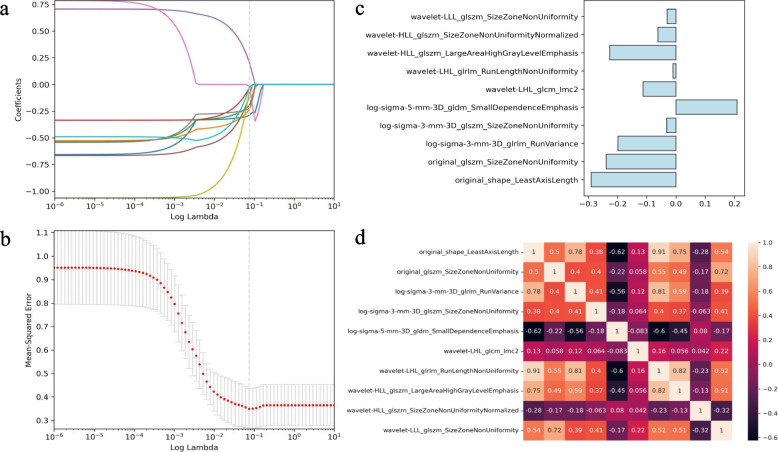
The selection process of the LASSO model. **a** Lasso coefficient profile plot with different log (λ) was shown. The vertical dashed lines represent 10 radiomics features with nonzero coefficients selected with the optimal λ value. **b** The LASSO model’s tuning parameter (λ) selection via minimum criterion. The vertical lines indicate the optimal value of the LASSO tuning parameter (λ). **c** Feature’s weights of selected 10 features. **d** Heatmap of 10 features

For manual tumor segmentation, good interobserver ICCs ranging from 0.80 to 0.89 and intraobserver ICCs ranging from 0.83 to 0.91 were obtained. A total of 1051 radiomics features were initially extracted from the 3D segmented VOI based on the portal venous phase. The analysis of variance performs initial feature screening to reduce the complexity of LASSO feature screening. 10 features with nonzero coefficients were selected through lasso regression (Table 3). Figure 3 illustrates the selection process of the LASSO model and the visualization of features. Finally, to prevent overfitting due to an excessive number of features, PCA was performed to reduce dimensionality, and features were finally reduced to 6.

**Table 3:** Lasso features’ selection results

ID	Radiomics’s feature name
1	original_shape_LeastAxisLength
2	original_glszm_SizeZoneNonUniformity
3	log-sigma-3-mm-3D_glrlm_RunVariance
4	log-sigma-3-mm-3D_glszm_SizeZoneNonUniformity
5	log-sigma-5-mm-3D_gldm_SmallDependenceEmphasis
6	wavelet-LHL_glcm_Imc2
7	wavelet-LHL_glrlm_RunLengthNonUniformity
8	wavelet-HLL_glszm_LargeAreaHighGrayLevelEmphasis
9	wavelet-HLL_glszm_SizeZoneNonUniformityNormalized
10	wavelet-LLL_glszm_SizeZoneNonUniformity

**Fig. 4:**
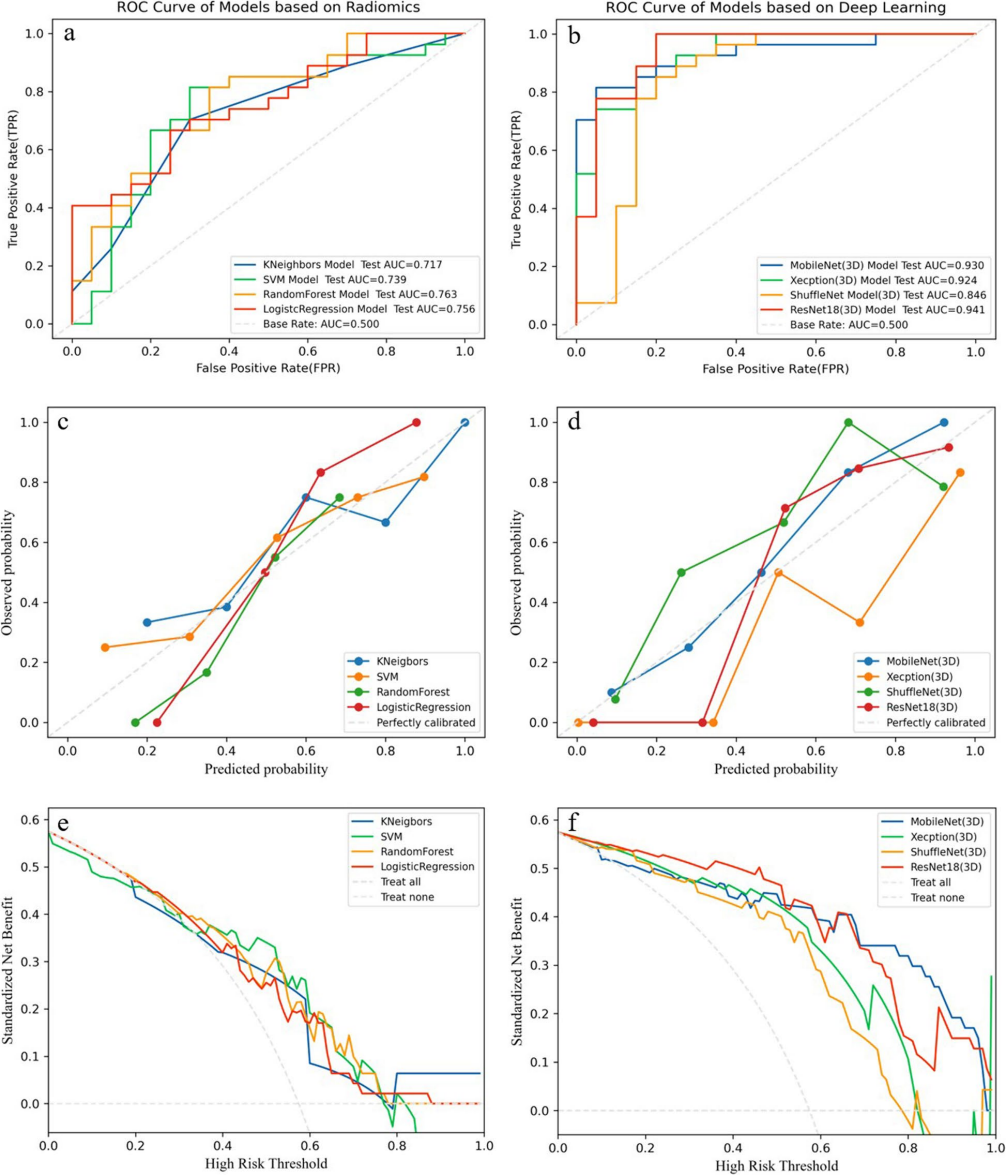
The ROC curves, calibration curves, decision curves among radiomics and deep learning groups, respectively. **a**, **c**, **e** ROC curves, calibration curves, and decision curves among radiomics models. **b**, **d**, **f** ROC curves, calibration curves, decision curves among deep learning models. The RF model and Resnet18 achieved the optimal efficiency in radiomics models and deep learning models, respectively. The calibration curves presented a good consistency between predicted and actual TSR in radiomics and deep learning models. The graphs show that the SVM model and ResNet18 have the greatest net benefit in radiomics models and deep learning models, respectively

In general, no matter in the training cohort or test cohort, deep learning models surpassed radiomic models. Specifically, in test cohort, deep learning models, including ShuffulNet, Xecption, MobileNet, and ResNet18, achieved AUCs of 0.846, 0.924, 0.930, and 0.941, respectively, outperforming radiomics models based on SVM, KNN, RF, and LR with AUCs of 0.739, 0.717, 0.763, and 0.756, respectively (Table 4, Fig. 4a, b). Furthermore, deep learning models exhibited higher accuracies: 0.830, 0.851, 0.872, and 0.894 for ShuffulNet, Xecption, MobileNet, and ResNet18, respectively, compared to 0.766, 0.702, 0.702, and 0.681 for radiomics models based on SVM, KNN, RF, and LR, respectively. Calibration curves demonstrated good calibration for both radiomics and deep learning models (Fig. 4c, d), however, radiomics models calibrated better than deep learning models. Decision curves indicated that the prediction models provided greater benefit than treating all or none of the patients, with deep learning models offering greater benefits than radiomics models (Fig. 4e, f). Additionally, we performed the DeLong test among eight models (Table 5). The results showed no significant difference was observed in four radiomics models alone or four deep learning models alone (all *p* > 0.05), whereas a significant difference was observed between radiomics models and deep learning models.

**Table 4:** The performance comparison of different models

Model	Category	Cohort	AUC (95% CI)	ACC	Precision	Recall	F1-Score
Clinical	T stage	Train	0.566 (0.477, 0.654)	0.550	0.694	0.284	0.402
		Test	0.610 (0.448, 0.772)	0.574	0.769	0.370	0.500
Radiomics	KNeigbors	Train	0.865 (0.832, 0.897)	0.785	0.784	0.785	0.784
		Test	0.717 (0.686, 0.757)	0.702	0.698	0.702	0.699
	SVM	Train	0.925 (0.908, 0.944)	0.847	0.847	0.847	0.847
		Test	0.739 (0.691, 0.791)	0.766	0.761	0.757	0.759
	Logistic regression	Train	0.859 (0.830, 0.886)	0.799	0.798	0.799	0.798
		Test	0.756 (0.719, 0.804)	0.681	0.673	0.670	0.671
	Random forest	Train	0.978 (0.970, 0.987)	0.889	0.891	0.889	0.888
		Test	0.763 (0.725, 0.802)	0.702	0.706	0.676	0.678
Deep learning	ShuffulNet	Train	1.000 (1.000, 1.000)	0.987	0.988	0.988	0.987
		Test	0.846 (0.816, 0.891)	0.830	0.826	0.826	0.826
	Xecption	Train	0.999 (0.999, 1.000)	0.987	0.988	0.988	0.987
		Test	0.924 (0.904, 0.940)	0.851	0.897	0.825	0.860
	MobileNet	Train	0.999 (0.999, 1.000)	0.988	0.988	0.988	0.987
		Test	0.930 (0.911, 0.951)	0.872	0.874	0.882	0.878
	ResNet18	Train	1.000 (1.000, 1.000)	0.998	0.998	0.998	0.998
		Test	0.941 (0.926, 0.962)	0.894	0.904	0.882	0.893

*AUC* area under the curve, *ACC* accuracy

**Table 5:** Comparison of ROC curves among different models by DeLong test

KNN/SVM	0.8313	SVM/LR	0.8927	LR/SN	0.3350	RF/Res	0.0206*
KNN/LR	0.7372	SVM/RF	0.8394	LR/Xec	0.0196*	SN/Xec	0.2722
KNN/RF	0.5784	SVM/SN	0.3140	LR/Mob	0.0276*	SN/Mob	0.2742
KNN/SN	0.1656	SVM/Xec	0.0534	LR/Res	0.0165*	SN/Res	0.1053
KNN/Xec	0.0106*	SVM/Mob	0.0368*	RF/SN	0.3752	Xec/Mob	0.9100
KNN/Mob	0.0051*	SVM/Res	0.0243*	RF/Xec	0.0238*	Xec/Res	0.6912
KNN/Res	0.0054*	LR/RF	0.9430	RF/Mob	0.0379*	Mob/Res	0.8273

*KNN* knearest neighbor, *SVM* support vector machine, *RF* random forest, *LR* logistic regression, *SN* ShuffulNetV2, *Xec* Xception, *Mob* MobileNetV3, *Res* ResNet18

^*^Represents *p* < 0.05

**Fig. 5:**
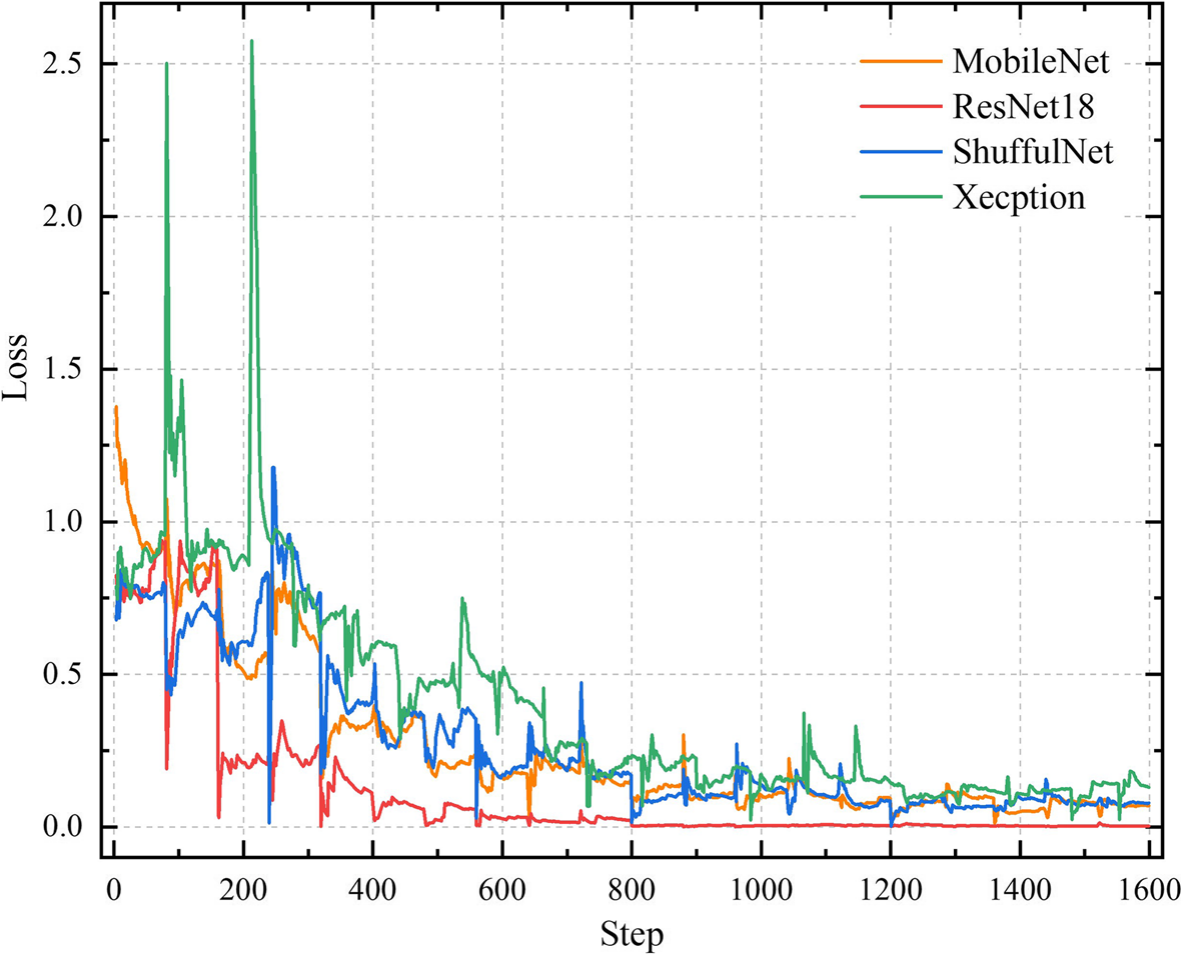
The loss values of various deep learning models in the training set showed fluctuation across different iteration steps

**Fig. 6:**
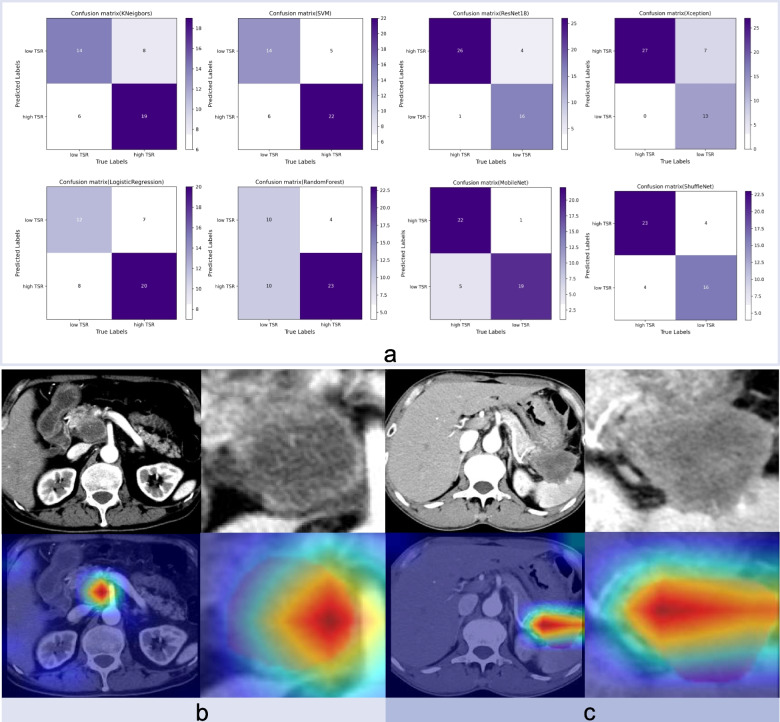
The confusion matrix of all models and original images and the corresponding gradient weighted class activation mapping (Grad-CAM) generated by ResNet18 of the representative patients. **a** The figure shows the number of patients in the test set who were correctly and incorrectly classified. **b** A 63-year-old man was diagnosed with pancreatic ductal adenocarcinoma (PDAC) with a high tumor stroma ratio (TSR). The tumor was in the pancreatic head. **b**, **c** Sixty-two-year-old man was diagnosed with PDAC with a low TSR. The tumor was in the pancreatic tail

The overall performance of Resnet 18 surpassed that of the other CNN models in the test cohort. Figure 5 displayed the training curves, and Resnet 18 exhibited the lowest loss value with the ability to minimize errors during training and showed faster convergence compared to any other CNN model tested. The specific network architecture of Resnet 18 is illustrated in Fig. 2b, with its most distinctive feature being the utilization of a residual network. Among all models evaluated, the ResNet18 model demonstrated the best diagnostic efficacy for this task. Figure 6a presents the confusion matrices of all models in the test cohort, revealing accurate predictions for 96.3% (26/27) of patients in the TSR-high group and 80% (16/20) of patients in the TSR-low group using the ResNet18 model. The Grad-CAM generated from ResNet18 provides a visual interpretation of the classified images, the ResNet18 model effectively highlighted the attention regions which contribute to classification decision within the samples (Fig. 6b). The darker the color is, the more focused the model is.

## Discussion

In our study, we aimed to compare the performance of automatic deep learning networks and radiomics models in differentiating TSR in patients with PDAC. Overall, our findings indicated that deep learning models outperformed radiomics models, with the ResNet18 model demonstrating the best performance. The models we developed and validated showed the potential for generalization, repeatability, and future clinical application.

In this study, we revealed that the TSR-low group had a significantly longer survival duration compared to the TSR-high group, suggesting a protective role of tumor stroma in the pathogenesis of PDAC. This finding is consistent with previous studies that have shown the impact of tumor stroma on tumor progression and prognosis [15, 16]. Additionally, studies by Torphy et al. also supported our findings by demonstrating a significant association between high stromal density and improved survival [8, 9]. Moreover, we observed higher T stages in the TSR-high group, which is consistent with the studies conducted by Meng et al., and Cai et al. [16, 33]. These findings collectively strengthen the understanding of the relationship between TSR and PDAC progression.

Previous studies have explored the correlation of imaging parameters with tumor stroma due to the comprehensive view provided by imaging scans and their ease of acquisition [34–36]. For instance, Mayer et al. demonstrated that the diffusion constant D from diffusion kurtosis imaging could be used as a non-invasive imaging biomarker to differentiate stroma-rich from stroma-poor tumors in PDAC [37]. CT imaging features have also been investigated by Cai et al. and Koay et al. as indicators of tumor stroma proportion in PDAC, with attenuation differences at the tumor-parenchyma interface showing potential for stratifying patients into prognostic subtypes [33, 35]. However, the afore-mentioned studies did not develop predictive models constructed by artificial intelligence technology.

In our study, we developed four radiomics and four deep learning models to compare their feasibility and effectiveness in CT-based TSR prediction. The AUCs achieved by our models ranged from 0.859 to 1.000 in the training group and 0.717 to 0.941 in the test group, surpassing previous similar research with an AUC of 0.93 in the training group and 0.63 in the validation group which only used XGBoost model based on radiomics model [16]. Our study had several advantages. Firstly, we collected data from three centers, ensuring dataset diversity and model generalization. Secondly, our end-to-end deep learning models automatically learned semantic and spatial features and eliminated the need for manually designed feature extraction, simplifying the process, and reducing the burden on doctors. This contrasted with traditional radiomics methods that required engineered features designed by humans. Lastly, our study highlighted the relatively poor generalizability of the radiomics model based on handcrafted features, as indicated by its lower sensitivity (ranging from 0.676 to 0.757) compared to the deep learning models (ranging from 0.825 to 0.882). In addition, radiomics models calibrated better than deep learning models in this study, we guessed the reason was due to traditional machine learning methods do well in small samples with diverse scanning protocols.

The lackluster performance across all four distinct radiomics models suggests that traditional radiomics features offer limited assistance in discerning high and low TSR. Notably, the random forest model outperforms the rest, which we attribute to its potency as a robust ensemble learning technique. By constructing numerous decision trees and amalgamating their predictions, the random forest effectively synthesizes forecasts from multiple machine learning models. Furthermore, its efficacy in diminishing overfitting through techniques like random feature selection and data sampling contributes to the model's enhanced generalization capabilities.

The notable superiority of all four deep learning models over traditional radiomics models suggests that this advantage arises from the deep learning models’ ability to extract features from three-dimensional medical images that better suit this specific medical image discrimination task. Unlike fixed and unchanging radiomics features, deep learning models can dynamically learn feature representations. The notable dissimilarity in feature expressions learned by deep learning models demonstrates the potential limitations of relying solely on conventional radiomics features. Among these models, ResNet18 outperforms the rest, and its exceptional performance solidifies ResNet18 as an exceptionally favorable choice for the specific task. This success can be attributed to its residual architecture enabling the network to capture features at varying scales and abstraction levels across different layers, thus enhancing the model's proficiency in representing features extracted from medical images.

Grad-CAM is a widely utilized post hoc interpretable technique applied to medical image research by using CNN. In the context of Grad-CAM, regions within the image displaying heterogeneous signals play a pivotal role in influencing the model’s prediction. The intensity of color within the Grad-CAM visualization denotes the level of significance and is attributed to these regions’ contribution to the model's final classification determination. Previous studies indicated these heterogeneous signals are often the regions of greater interest in clinical work [38, 39]. Additionally, it primarily focused on the boundary and internal regions of the tumor, the blood vessels, bones, and normal pancreatic parenchyma adjacent to tumor regions did not exhibit significant activation, demonstrating its ability to ignore non-core areas for analysis.

However, our study had some limitations. First, we excluded patients who received antitumor therapy before surgery, which might have introduced selection bias. Because uniform selection standard for patients’ therapy management contributes to avoid confounding influence on the survival time of PDAC except for tumor stroma. We speculated that patients who received antitumor therapy (radiotherapy, chemotherapy, chemoradiotherapy) before surgery may affected the pathological observation on TSR, so we strict screening criteria in this study. In the future, we will enroll more cases including patients with and without antitumor therapy before surgery to investigate the role of TSR from a more comprehensive perspective, in addition, we will collect patients only with antitumor therapy before surgery to complete subgroup analysis. Second, our study was retrospective and the evaluation of TSR goes beyond routine clinical needs, resulting in a limited quantity of sample data and potential mild overfitting. However, for the radiomics models, we employed feature dimensionality reduction techniques such as PCA and fine-tune hyperparameters to prevent overfitting and mitigate model complexity. Additionally, an ensemble learning approach such as RF was adopted to combine multiple decision tree models and mitigate the impact of overfitting on individual trees. Within deep learning models, we introduced data augmentation techniques on the training dataset, involving rotations, translations, and scaling, to augment the diversity of medical images and enhance the model's ability to generalize. Moreover, regularization techniques were employed by incorporating regularization terms within both the model architecture and loss function to prevent overfitting. Lastly, we implemented dropout on the model's classifier, randomly deactivating a fraction of neurons by setting them to zero, thereby reducing complex co-adaptations between neurons and aiding in overfitting prevention. In general, we leveraged cross-validation techniques to partition the limited data into multiple subsets for model training and validation. This approach maximizes data utilization and yields a more reliable estimation of model performance. Furthermore, by utilizing pre-trained models, we transferred knowledge from other data sources to the constrained medical image dataset, effectively enhancing the overall model performance. Third, we trained deep learning models using original abdominal images instead of segmented tumor VOI, which may cause interference from underlying background factors; however, the use of grad-cam revealed that attention regions were predominantly focused on the tumor itself, guaranteeing efficiency and accuracy of the model’s performance.

In conclusion, non-invasive assessment of stroma proportion provides a feasible approach for stratifying patients with distinct clinical outcomes in PDAC. Deep learning, as a quantitative method, shows promising performance in predicting poor prognosis compared to the traditional radiomics workflow. Therefore, preoperative TSR prediction offers new insights into the diagnosis and treatment of this lethal disease.

## Data Availability

The datasets used or analyzed during the current study are available from the corresponding author on reasonable request.

## Abbreviations

3D-VOI: Three-dimensional volume of interest
ACC: Accuracy
AUC: Area under the curve
CA19-9: Carbohydrate antigen 19–9
CEA: Carcinoembryonic antigen
CNN: Convolutional neural network
DCA: Decision curve analysis
FFPE: Formalin-fixed paraffin-embedded
GLCM: Gray level cooccurrence matrix
GLDM: Gray level dependence matrix
GLRLM: Gray level run length matrix
GLSZM: Gray level size zone matrix
Grad-CAM: Gradient-weighted class activation mapping
IBSI: Image Biomarker Standardization Initiative
ICC: Intraclass correlation coefficient
KNN: Knearest neighbor
LR: Logistic regression
NGTDM: Neighboring gray tone difference matrix
OS: Overall survival
PCA: Principal component analysis
PDAC: Pancreatic ductal adenocarcinoma
RF: Random forest
ROC: Receiver operating characteristic curves
ROI: Region of interest
SVM: Support vector machine
TBIL: Total bilirubin
TME: Tumor microenvironment
TSR: Tumor-stroma ratio
